# Differences of respiratory kinematics in female and male singers – A comparative study using dynamic magnetic resonance imaging

**DOI:** 10.3389/fpsyg.2022.844032

**Published:** 2022-12-05

**Authors:** Louisa Traser, Carmen Schwab, Fabian Burk, Ali Caglar Özen, Michael Bock, Bernhard Richter, Matthias Echternach

**Affiliations:** ^1^Institute of Musicians’ Medicine, Faculty of Medicine, Medical Center – University of Freiburg, Freiburg, Germany; ^2^Faculty of Medicine, University of Freiburg, Freiburg, Germany; ^3^Department of Prosthetic Dentistry, Center for Dental Medicine, Faculty of Medicine, Medical Center – University of Freiburg, Freiburg, Germany; ^4^Department of Phoniatrics and Pediatric Audiology, University Medical Center Münster, Münster, Germany; ^5^Department of Radiology, Medical Physics, Medical Center – University of Freiburg, Faculty of Medicine, University of Freiburg, Freiburg, Germany; ^6^Division of Phoniatrics and Pediatric Audiology, Department of Otorhinolaryngology, Munich University Hospital (LMU), Munich, Germany

**Keywords:** respiration, phonation, singer, diaphragm, voice, sex, gender, dynamic magnetic resonance imaging

## Abstract

Breath control is an important factor for singing voice production, but pedagogic descriptions of how a beneficial movement pattern should be performed vary widely and the underlying physiological processes are not understood in detail. Differences in respiratory movements during singing might be related to the sex of the singer. To study sex-related differences in respiratory kinematics during phonation, 12 singers (six male and six female) trained in the Western classical singing tradition were imaged with dynamic magnetic resonance imaging. Singers were asked to sustain phonation at five different pitches and loudness conditions, and cross-sectional images of the lung were acquired. In each dynamic image frame the distances between anatomical landmarks were measured to quantify the movements of the respiratory apparatus. No major difference between male and female singers was found for the general respiratory kinematics of the thorax and the diaphragm during sustained phonation. However when compared to sole breathing, male singers significantly increased their thoracic movements for singing. This behavior could not be observed in female singers. The presented data support the hypothesis that professional singers follow sex-specific breathing strategies. This finding may be important in a pedagogical context where the biological sex of singer and student differ and should be further investigated in a larger cohort.

## Introduction

Effective respiratory control is an important factor of voice production and its dysbalance has been associated with several voice disorders (Hixon and Hoit, 2005). However, in voice pedagogy very different descriptions can be found for what is considered an efficient breathing strategy, and a wide variation of respiratory movement patterns can be observed in successful singers during Western style classical singing (McCoy, 2005; Sonninen et al., 2005). These differences could be related to the singers’ sex, but studies analyzing sex-related influences on respiratory kinematics during voice production so far showed conflicting results (Watson and Hixon, 1985; Watson et al., 1990; McCoy, 2005): a questionnaire-based survey reports significant differences in the localization of breathing motions between female (lower abdomen) and male singers (upper abdomen). McCoy describes that these differences correlated well with the sex of authors of relevant singing pedagogic literature and their descriptions on where respiratory movements should take place during singing (McCoy, 2005a). However, a degree of mismatch between the believed and measured respiratory movements are described even for professional singers (Watson and Hixon, 1985; Griffin et al., 1995). In measurements by Watson and colleagues of the anterior–posterior diameter of the chest and the abdomen during singing, the authors were not able to experimentally confirm the sex-related difference postulated by McCoy (Watson and Hixon, 1985; Watson et al., 1990). Still, a recent study analyzed ventilation profiles of professionally trained singers and suggested sex-related difference in the movement pattern of thorax and diaphragm (Traser et al., 2017a). With dynamic MRI respiratory movements can be imaged dynamically during phonation. Dynamic MRI was first used in a pilot study on six professional singers to compare sustained phonation with exhalation (Traser et al., 2017b). General aspects of breath support during singing were here firstly described by specific respiratory kinematics of the thorax and diaphragm during sustained phonation.

The aim of this study is the analyzation of respiratory movements during phonation with regards to sex-related differences. A better understanding of possible sex-specific differences in respiratory movements during singing could help to avoid counterproductive or even vocal health threatening habits in singing voice training.

MRI was chosen as imaging technique as it provides a radiation-free, non-invasive way to record dynamic physiological processes inside the human body. It is therefore possible to analyze inter-individual differences in respiratory kinematics as well as respective changes of lung volume related anatomical distances in individuals including healthy ones. Still, beside the pulmonary aspects during singing it is also important to acquire information about the processes in the other parts of the voice instrument (e.g., vocal fold vibration, subglottic pressure, and resonance properties) as these systems are always interdependent (Sundberg, 1987). Nevertheless, due to the magnetic forces and the loud noise during MR scanning, it is necessary to record supplementary data outside the scanner in a separate session as described in detail in the method section. Only electroglottography (EGG) could be successfully implemented synchronously to MRI recording (Özen et al., 2015).

## Materials and methods

### Subjects

This study was approved by the institutional review board (Medical Ethics Committee) of the University of Freiburg, Germany (273/14). Twelve singers trained in the Western classical singing tradition were included, of which 10 were professional singers, defined as having their primary income from singing, and two subjects (Nr. 6 and 7) were experienced semi-professional singers with more than 15 years of singing training who regularly sing in semi-professional ensembles. All professional trained singers were graduates from different music universities. Here they were trained by different singing teachers in the Western classically singing technique. The semi-professional singers did not graduate from music university but had received regular singing lessons for more than 15 years from professionally trained singing pedagogues. Table 1 lists age, sex, voice classification, classification according to the Bunch and Chapman taxonomy^1^ (Bunch and Chapman, 2000) and subject characteristics including vital capacity (VC), forced expiratory volume in 1 s (FEV1), body height and weight and body mass index (BMI) of all subjects. At the time of the study none of the participants reported vocal diseases, histories of voice disorders, or respiratory pathologies. Spirometry was performed in all subjects to analyze pulmonary function using a spirometer (ZAN, Messgeräte GmbH, Oberthulba, Germany) according to current clinical guidelines (Sorichter, 2009).

**Table 1:** Subject number, age, sex, voice classification, classification according to the Bunch and Chapman taxonomy (Bunch and Chapman, 2000), vital capacity (=VC), forced expiratory volume in 1 s (= FEV1), body height and body-mass-index (=BMI).

Subject	Age	Sex	Voice classification	Bunch and Chapman taxonomy	VC in l	FEV1 in l/s	Height in cm	BMI in kg/m^2^
1	25	Female	Soprano	3.15b1	3.87	3.46	167	19.72
2	28	Female	Soprano	4.5	4.72	3.88	165	23.87
3	25	Female	Soprano	3.15b1	3.71	3.28	158	18.82
4	34	Male	Tenor	3.4	5.10	3.35	175	21.55
5	32	Female	Soprano	3.1b	3.28	2.90	157	21.50
6	33	Male	Tenor	5.4	4.92	3.21	171	21.20
7	32	Female	Soprano	5.4	4.56	3.77	170	22.49
8	25	Male	Tenor	7.2	5.11	4.15	186	22.25
9	27	Male	Tenor	3.1b	5.13	4.11	172	27.04
10	42	Male	Tenor	4.5	6.32	5.31	192	25.22
11	30	Male	Baritone	3.1a	6.26	5.29	190	27.70
12	33	Female	Soprano	3.4	3.19	2.29	162	26.67

Subjects 1–3, 10, 11 were also part of the pilot study (Traser et al., 2017b).

### Magnetic resonance imaging

**Figure 1:**
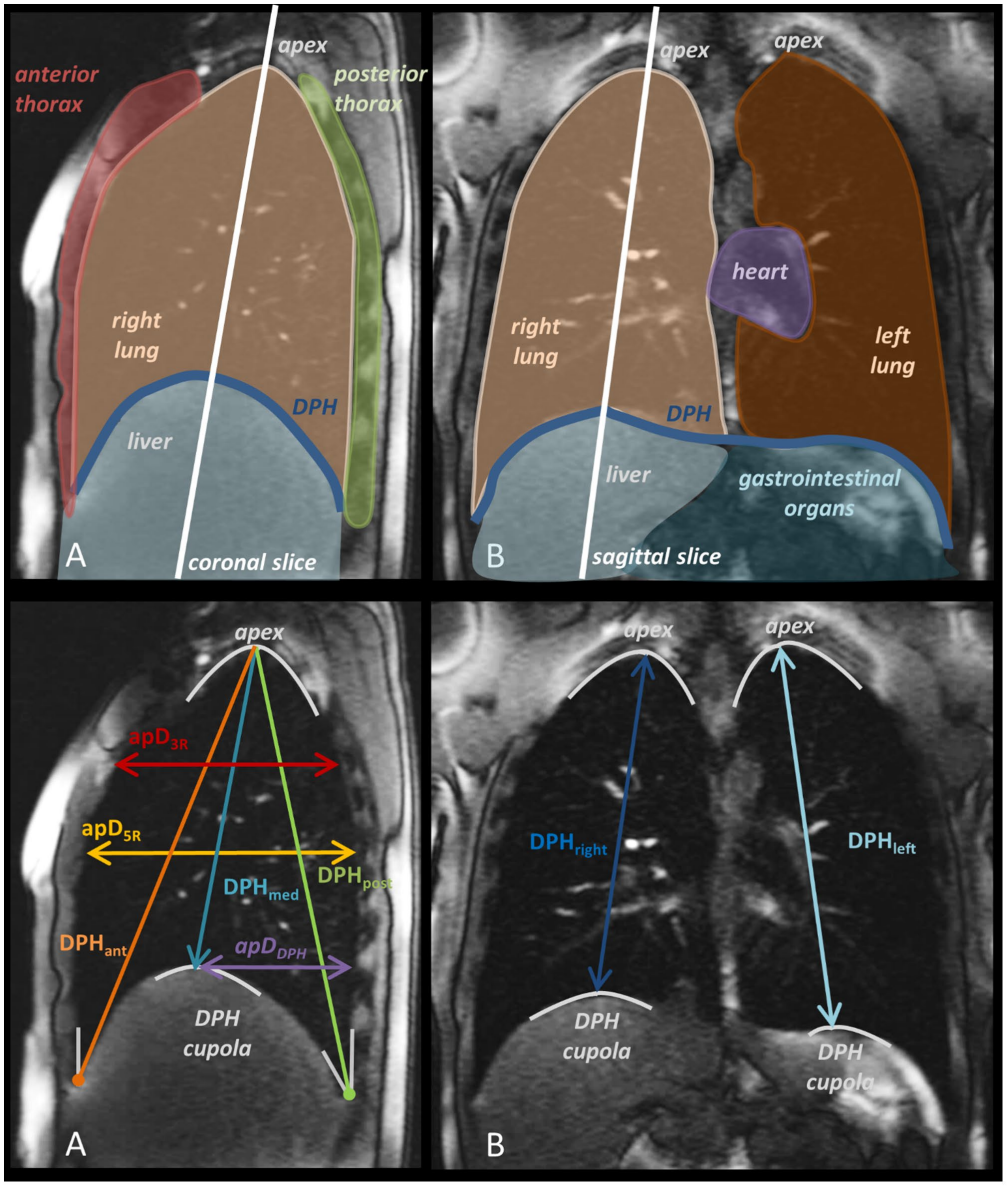
Sagittal **(A)**, and coronal **(B)**, MR image with anatomical designations and slice orientation (upper row) and measured distances with their definition according to anatomical landmarks (lower rows).

The imaging of the singers’ breathing apparatus was performed using a clinical 1.5 T MRI system (Tim Symphony, Siemens, Erlangen, Germany) as reported in the pilot study (Traser et al., 2017b). The subject positioning was supine (see limitation section for detailed discussion of the potential effects of gravity on the results). Dynamic imaging was done using a 2D trueFISP imaging sequence (repetition time/echo time = 3/1.5 ms, α = 6°, bandwidth (BW) = 977 Hz/px, slice thickness = 10 mm, acquisition matrix = 256, field of view (FOV) = 420 mm) with a temporal resolution of approximately three frames per second. First, localizer images at three different planes were acquired to define the imaging planes in sagittal and coronal orientations. For the sagittal trueFISP images, a slice through the right lung was chosen to avoid artefacts caused by heart motion, which would complicate the image analysis. The sagittal plane was selected so that the vertex of the DPH cupola and the apex of the lung could be identified (see Figure 1 for details). The coronal plane was chosen similarly, encompassing both vertices of the left and right DPH cupola and the apices of the left and right lung. During imaging the subjects wore headphones for hearing protection and communication.

### Tasks

During the MRI, singers were first ask to breathe in and out as deeply as possible in order to assess the movements of their respiratory system during VC breathing. As in the pilot study (Traser et al., 2017b), singers were asked to sustain phonation on vowel [a:] at three different pitches for as long as possible (maximum phonation time, MPT) at medium loudness (mezzo forte, mf). The subjects were then asked to repeat phonation at the medium pitch in two additional loudness conditions (pianissimo, pp.; fortissimo, ff). The phonation tasks were chosen according to the voice classification of the singer and they represented a low (P_1_), medium (P_2_), and high pitch (P_3_) in the tessitura of the respective repertoire of the singer (see Table 2 for details). The tasks were chosen to allow evaluation not only of a single frequency range but to provide a basic representation at different points of the voice range profile. First, all dynamic MRI acquisitions were performed in a sagittal image orientation, and then the entire protocol was repeated in a coronal orientation. The tasks were explained to the singers prior to the experiment and they had the possibility to repeat a task if the singer was not satisfied with the performance.

**Table 2:** List of the tasks performed by the subjects according to different voice classifications including pitch and loudness.

	Female		Male			
Voice classification/task	Soprano		Tenor		Baritone	
Vital capacity breathing	Maximal inspiration and expiration					
	Pitch	Loudness	Pitch	Loudness	Pitch	Loudness
P1mf:	A3 (220Hz)	mf	A2 (110Hz)	mf	F2 (87 Hz)	mf
P2mf:	A4 (440Hz)	mf	A3 (220Hz)	mf	F3 (175Hz)	mf
P3mf:	A5 (880Hz)	mf	A4 (440Hz)	mf	F4 (349Hz)	mf
P2pp:	A4 (440Hz)	pp	A3 (220Hz)	pp	F3 (175Hz)	pp
P2ff:	A4 (440Hz)	ff	A3 (220Hz)	ff	F3 (175Hz)	ff

Data of the two semi-professional singers were collected as part of a different study protocol, which did not include VC breathing. Thus, their data could only be analyzed during phonation and was excluded for evaluation in relation to VC breathing. A total of 12 image series (six sagittal and six coronal) was acquired per subject (five image series for the two singers with the missing VC data).

### Image analysis

In each dynamic MRI frame, distances between anatomical landmarks were measured using an in-house software developed in Matlab 9.1 (MathWorks, Natick, Massachusetts, United States). The dynamic changes of these distances during phonation allow comparing respiratory motion patterns in the different areas of the respiratory system. Six distances were derived from the sagittal images: anterior diaphragm height (DPH_ant_), medial diaphragm height (DPH_med_), posterior diaphragm height (DPH_post_), anterior–posterior diameter at the height of the 3^rd^ rib (pD3R), and anterior–posterior diameter at the height of the 5^th^ rib (apD5R). From the coronal image series two parameters were extracted: medium diaphragm height of the right lung (DPH_right_) and medium diaphragm height of the left lung (DPH_left_). A detailed graphical description of distances and anatomical aspects in the images are shown in Figure 1. Measurement points are defined in Table 3. For best comparability these measures were selected based on existing literature on the subject (Traser et al., 2017b, 2020a, 2021).

**Table 3:** Anatomical definition of distances in sagittal and coronal plane.

**Sagittal plane**	
DPH_ant_	Craniocaudal lung height from the angle of the anterior DPH and the RC to the apex of the lung
DPH_med_	Craniocaudal lung height from the medial part DPH at its highest point of the cupola to the apex of the lung
DPH_post_	Craniocaudal lung height from the angle of the posterior DPH and the RC to the apex of the lung
apD_3R_	The anterior–posterior lung diameter at the height of the 3th rip
apD5_R_	The anterior–posterior lung diameter at the height of the 5th rip
apD_DPH_	The anterior–posterior diameter from the highest point of the cupola of DPH to the posterior boundary of the lung
**Coronal plane**	
DPH_right_ and DPH_left_	Craniocaudal lung height from highest point of DPH to the apex of the right and left lung

DPH = diaphragm.

### Data workflow and normalisation

To compare sustained phonation of different durations, the time axis was re-scaled: the beginning of phonation was defined as *t*_start_ and the end as *t*_end_. The measured distances (*A*) at different timepoints A(t) and different locations were normalized (*A*_norm_) according to


$$A_{norm}\left(t\right)=\frac{A\left(t\right)-A\left(t_{end}\right)}{A\left(t_{start}\right)-A\left(t_{end}\right)}\cdot 100\%$$

Additionally, the slope (*m)* of all graphs was calculated in steps of 20% as the ratio of changes in measured distances over time (*m*_1_ to *m*_5_).


$$m_{n}=\frac{A_{norm}\left(t_{n}\right)-A_{norm}\left(t_{n-1}\right)}{t_{n}-t_{n-1}}$$

In the next step, data were normalized according to vital capacity at maximum and minimum respiration. Here, the amplitude at maximal inspiration was set to 100% *(A_VCmax_*), and the amplitude at maximal expiration was set to 0% *(A_VCmin_*). Again, phonation time was scaled as described above. Based on the individual normalization to VC breathing, a quotient was established that represents the movement range during maximum sustained phonation in relation to the individual’s VC respiration (range of movement, ROM_p/r_). This was calculated from the raw data according to:


$$ROM_{p/r}=\frac{A\left(t_{start}\right)-A\left(t_{end}\right)}{A\left(t_{VCmax}\right)-A\left(t_{VCmin}\right)}\cdot 100\%.$$

### Electroglottography and audio recording

To characterize the singing performance, additionally vocal fold contact and fundamental frequency was measured during dynamic lung MRI using an MRI-compatible electroglottography (EGG) system (Özen et al., 2015; Laryngograph Ltd. London, United Kingdom). EGG recordings were used to monitor the vibratory cycles of vocal folds and calculate the open quotient (OQ), i.e., the proportion of time within a cycle that the vocal folds are not in contact. OQ was calculated according to Howard et al. (Howard et al., 1990; Howard, 1995) combining an EGG-based threshold method for detection of glottal opening (at 3/7) with detection of glottal closing instants in the dEGG (derivative of EGG) signal. Additionally, the EGG signal was used to determine the fundamental singing frequency (*f*_o_). The mean value of *f*_o_ was calculated for a stable phonation segment of 1 s. Deviations from the expected *f*_o_ were calculated in cents due to its logarithmic scale. The audio signal was also simultaneously recorded using a microphone system recorded at 30 cm from the mouth (Pre-polarized Free-field 1/2″ Microphone, Type 4,189, Brüel&Kjær, Nærum, Denmark).

### Pressure and sound pressure level measurements

To determine the subglottic pressure *p_sub_*, each subject repeated the performance of the tasks in section “Tasks” outside of the MRI in a supine position. *P_sub_* was measured in a separate sound treated room directly before the MRI measurement and was determined from the oral pressure during the/p/−occlusions according to Baken and Orlikoff (2000). Oral pressure was measured *via* a short plastic tube (inner diameter: 1.5 mm) mounted in a Rothenberg mask that was placed in the right-hand corner of the subject’s mouth, and which was connected to a pressure transducer (PT-70, Glottal Enterprises, Syracuse, United States). The audio and EGG recordings were also performed during *p_sub_* measurements. The audio signal was recorded at a distance of 1 m from the mouth by a omnidirectional microphone (Laryngograph Ltd. London, United Kingdom) to calculate the sound pressure level (SPL). A calibration of the SPL was performed prior to each measurement using a sound level meter (Sound level meter 331, Tecpel, Taipe, Taiwan). *P_sub_* and SPL were analyzed using the Aeroview software (Version. 1.4.5, Glottal Enterprises, 2010, Syracuse, United States). Due to technical problems *p_sub_* and SPL data from subject 6 had to be excluded from the analysis.

### Statistics

Sex-related differences of the gradients of normalized movement curves were analyzed using a repeated-measures ANOVA (rmANOVA) by including five timesteps of a curve progression (m_1−5_, = levels in rmANOVA = 5). As an overview, first all data were initially included in one calculation with covariates (subject, task and location) and factor: sex. The Greenhouse–Geisser correction was used in order to correct the degrees of freedom. In a second step separate rmANOVA calculations were performed for each location and each task separately again with factor sex and Greenhouse–Geisser correction.

Sex-related differences of ROM_p/r_ were assessed using a univarite ANOVA test. Correlation analysis between OQ, *p_sub_* and SPL was performed using a two-tailed Spearman-rho correlation (*r_s_* = 0.2–0.5: low to medium correlation, *r_s_* = 0.5–0.8: substantial correlation, *r_s_* = 0.8–1.0: high to perfect correlation). For all statistical analyses, SPSS 23.0 software (SPSS, Inc., Chicago, IL) was used. The level of significance was set to *p* < 0.05.

## Results

### Study cohort characteristics

**Figure 2:**
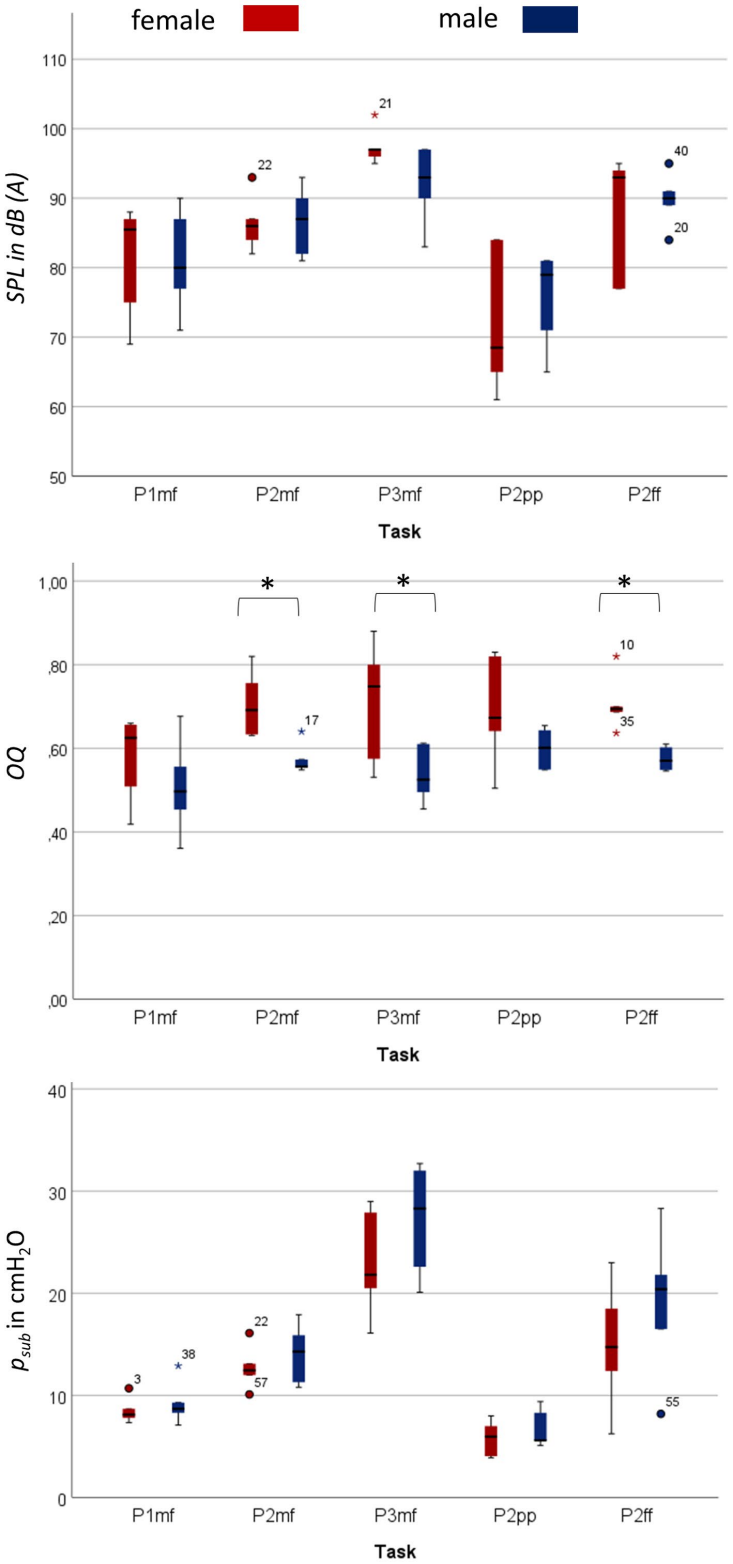
Significantly larger open quotient (OQ) for female compared to male singers in the respective task, no significant difference for subglottic pressure (*p_sub_*) and sound pressure level (SPL). The figure shows boxplots of OQ, *p_sub_* and SPL for each task separately for male and female singers. An increase in *p_sub_* can be seen from the lowest to highest pitch (P1 mf to P2 mf to P3 mf) as well as from the lowest to the highest loudness condition (P2 pp to P2 mf to P2 ff). The same trajectory can also be seen for the measured SPL. No significant difference is found between male and female singers. In contrast, while OQ increases with pitch in female singers, no pitch-dependent relationship was found for male singers. Significant differences are marked with *. Details on statistic evaluation can be found in Supplementary Table S1.

Female singers were significantly smaller and had a lower VC compared to male singers, but there was no statistical difference between the BMI of men and women (see Table 4). Boxplots in Figure 2 give an overview of mean OQ, SPL and *p_sub_* for male and female singers’ different tasks: OQ correlated significantly with *f*_o_ (*r* = 0.38, *p =* 0.04) in female singers with a higher mean OQ with increasing *f*_o_. In contrast, no significant correlation was found between OQ and *f*_o_ for male singers (*r* = 0.05, *p =* 0.80). Additionally no significant correlation was found for either male or female singers between SPL and OQ (female: *r* = −0.32, *p =* 0.19; male: *r* = −0.14, *p =* 0.62). On average across all tasks, female singers phonated with a larger OQ compared to male singers [mean OQ female/male = 0.68 ± 0.02/0.56 ± 0.1; F(1/59) = 25.68, *p* = <0.001, *ƞ^2^* = 0.32]. Each task was also evaluated individually (see Supplementary Table S1): OQ was significantly higher for female compared to male singers for P2mf, P3mf, and P2ff but not for P1mf and P2pp.

**Table 4:** Mean female and male body-mass-index (BMI), vital capacity (VC) and body height including standard error and the results of analysis of statistical differences (significant differences are marked in darker grey).

Parameter	Mean female (Standard error)	Mean male (Standard error)	Statistical difference
BMI	22.18 (0.82)	24.16 (0.83)	*F*(1;11) = 1.44, *p* = 0.26, *ƞ^2^* = 0.13
VC	3.89 (0.18)	5.47 (0.18)	*F*(1;11) = 18.55, *p* = 0.002*, *ƞ^2^* = 0.65
Body height	163.17 (1.47)	181.00 (2.72)	*F*(1;11) = 16.60, *p* = 0.002*, *ƞ^2^* = 0.62

*P_sub_*, both as an overall average and for each task separately (see Supplementary Table S1), did not differ significantly between female and male singers [mean *p_sub_* female/male = 12.96 ± 1.24/ 15.26 ± 1.55 mmH_2_0; F(1/59) = 1.24, *p* = 0.27, *ƞ^2^* = 0.02]. Significantly higher *p_sub_* occurred with higher *f*_o_ (female: *r* = 0.60, *p <* 0.001; male: *r* = 0.69, *p <* 0.001) and higher SPL (female: *r* = 0.76, p < 0.001; male: *r* = 0.70, *p* = 0.004) for both female and male singers.

Similarly, mean SPL as well as SPL of each task (See Supplementary Table S1) did not differ significantly between female and male singers [mean *SPL* female/male = 85.10 ± 1.96/84.96 ± 1.51 dB; F(1/59) = 0.002, *p* = 0.97, *ƞ^2^* < 0.001], although significantly higher SPL correlated with higher *f*_o_, for both female and male singers (female: *r_s_* = 0.55, *p* = 0.002; male: *r_s_* = 0.50, *p* = 0.01).

The maximum phonation time (MPT) was significantly longer for male compared to female singers [*F*(1,119) = 39.9, *p* = <0.001, *ƞ^2^* = 0.28].

### Respiratory kinematics during sustained phonation

**Figure 3:**
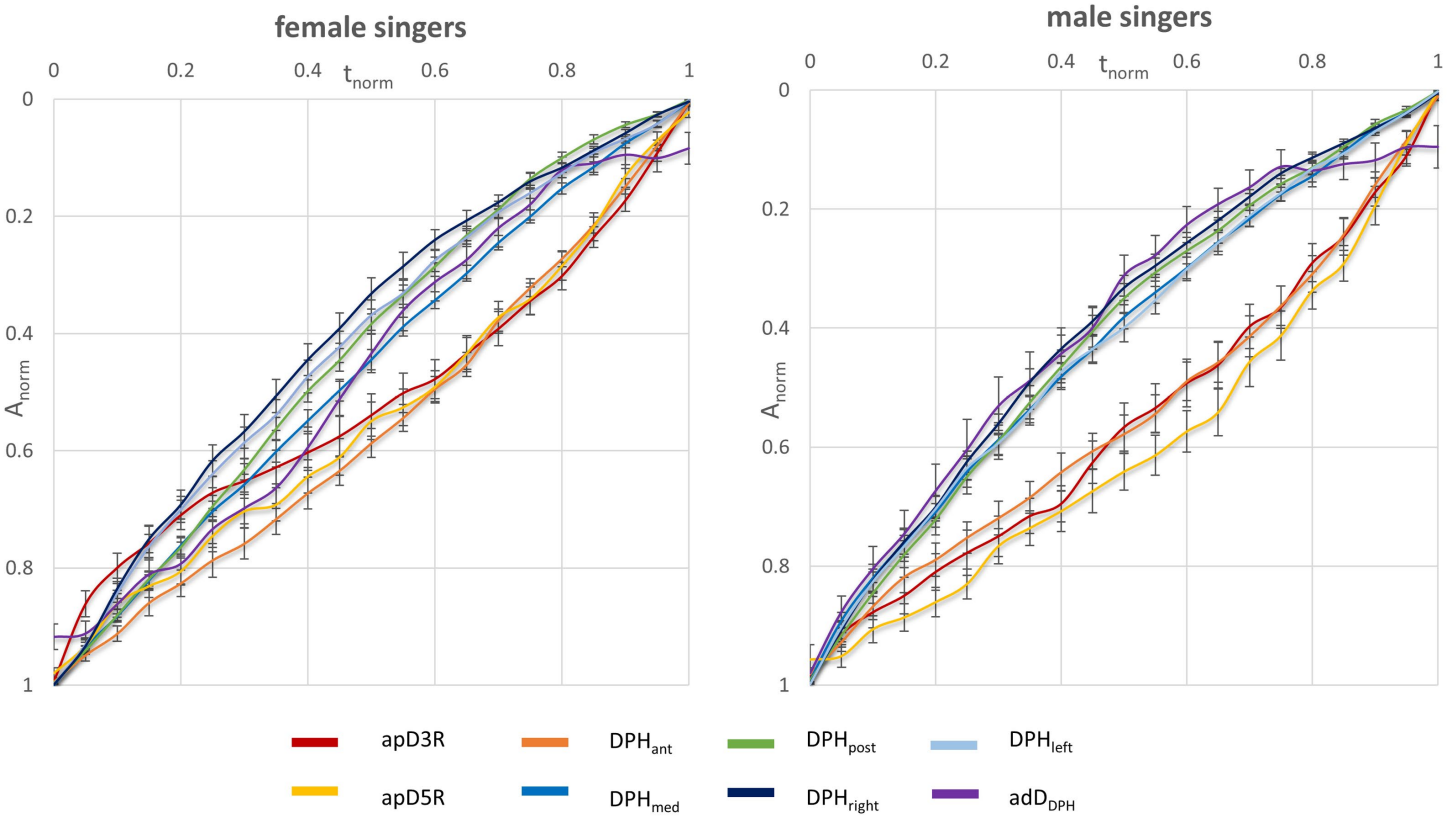
No major difference in movement velocities during sustained phonation between female and male singers. The figure displays the normalized amplitude (*A_norm_*) of the mean movement curves for different locations for male (right) and female singers (left) as a function of normalized phonation time.

Respiratory kinematics of diaphragm and thorax show no major difference between the normalized movement curves of male and female singers for all measured locations (Figure 3): The motion of the posterior and mid part of the DPH is quicker during the first half of maximum phonation time and slower at the end, while that of the anterior DPH and the thorax distances is slower in the beginning and quicker toward the end. rmANOVA of the curve gradient showed no statistically significant sex-related difference in curve progression [*F*(4,472) = 1.94, *p* = 0.10, *ƞ^2^* = 0.02]. Still, subgroup analysis of each location separately revealed a difference in progression of apD3R: While this distance decreased rapidly in the first 20% of phonation time in female singers (mean gradient in m_1_ = −1.50), male singers held it more consistently (mean gradient in m_1_ = −0.73). In contrast, the posterior movement of the DPH cupola (apDDPH) was faster in the first 20% of the phonation time in male singers (mean gradient in m_1_ = −1.50) and held more consistently in female singers (mean gradient in m_1_ = −0.66).

### Comparison with VC breathing

**Figure 4:**
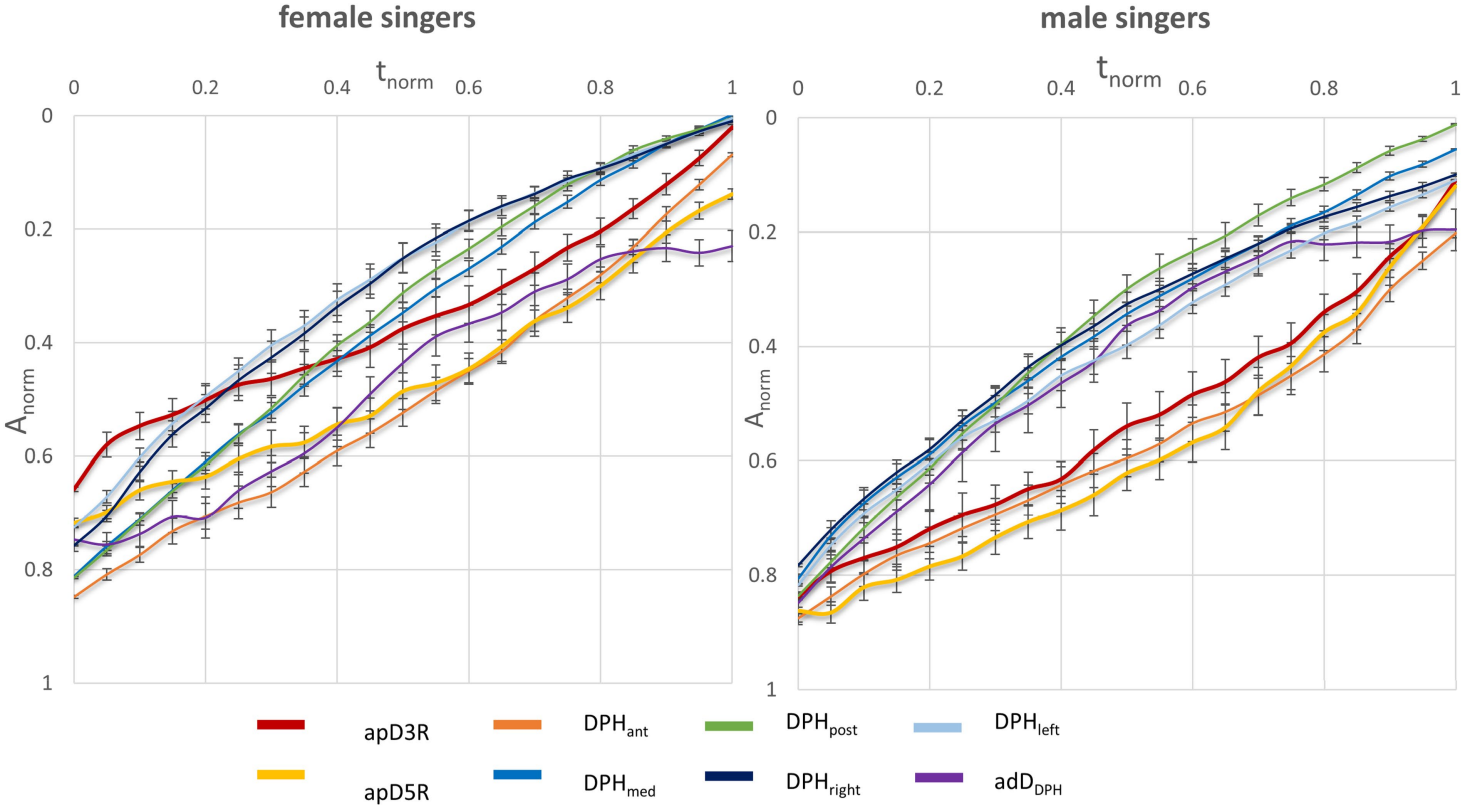
Female singers started at lower thoracic vital capacity levels compared to male singers. Mean movement curves of female (left) and male (right) normalized to vital capacity inspiration and expiration are displayed including standard error bars. The x-axis displays normalized maximum phonation time (*t_norm_*) and the y-axis displays the normalized curve amplitude (*A_norm_*) as a percentage of individual maximum inspiration and expiration. Each location of the respiratory system is displayed in a separate curve.

**Figure 5:**
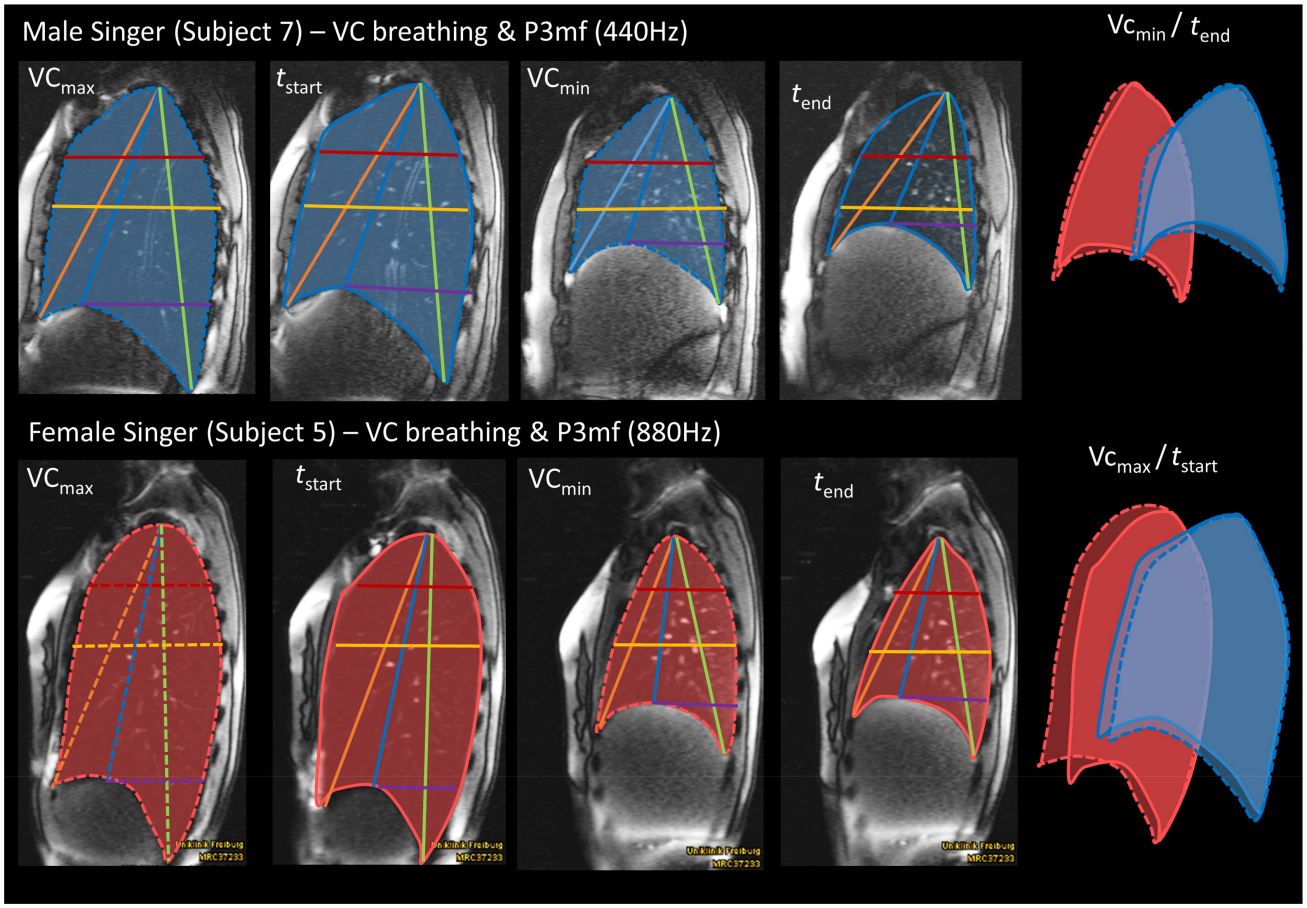
Exemplary illustration of a female singers’ reduction and male singers accentuation of thorax elevation for phonation compared to vital capacity breathing. The figure displays MR images of an example male (10) and female (5) subject during maximum inhalation (*VC_max_*), maximum exhalation (*VC_min_*), start of sustained phonation (*t_start_*) and end of sustained phonation (*t_end_*). A corresponding pitch (P3 mf, 880 Hz in female/440 Hz in male) was chosen. The superimposed lung cross-sections of a sagittal MR image illustrate that the female singer reduced her thoracic inhalation movement for phonation, while it was increased by the male singer.

In the next step the kinematic data was analyzed in relation to the individual respiratory behavior during VC breathing. Figure 4 displays the respiratory kinematics in relation to each singers’ maximum inspiration and expiration. While on average, male singers started to phonate at 87% of their maximal thorax expansion, female singers began phonation between 65% (apD3R) to 75% (apd5R) of theirs. Figure 5 displays an example of the lung configuration in a sagittal image of a female (#5) and a male subject (#10). Here, a difference between the inspiratory behavior during singing and breathing can be inspected visually: The male subject increased his thorax movement during singing, while the female subject decreased it. Another dynamic example is given in a Supplementary Video (Supplementary Video S3). Here synchronized respiratory kinematics during inspiration and phonation of one female and one male singer are shown in a dynamic MRI video of the right lung in a sagittal slice. The video is played three times, in the last version at half speed, inspiration and phonation are marked.

**Figure 6:**
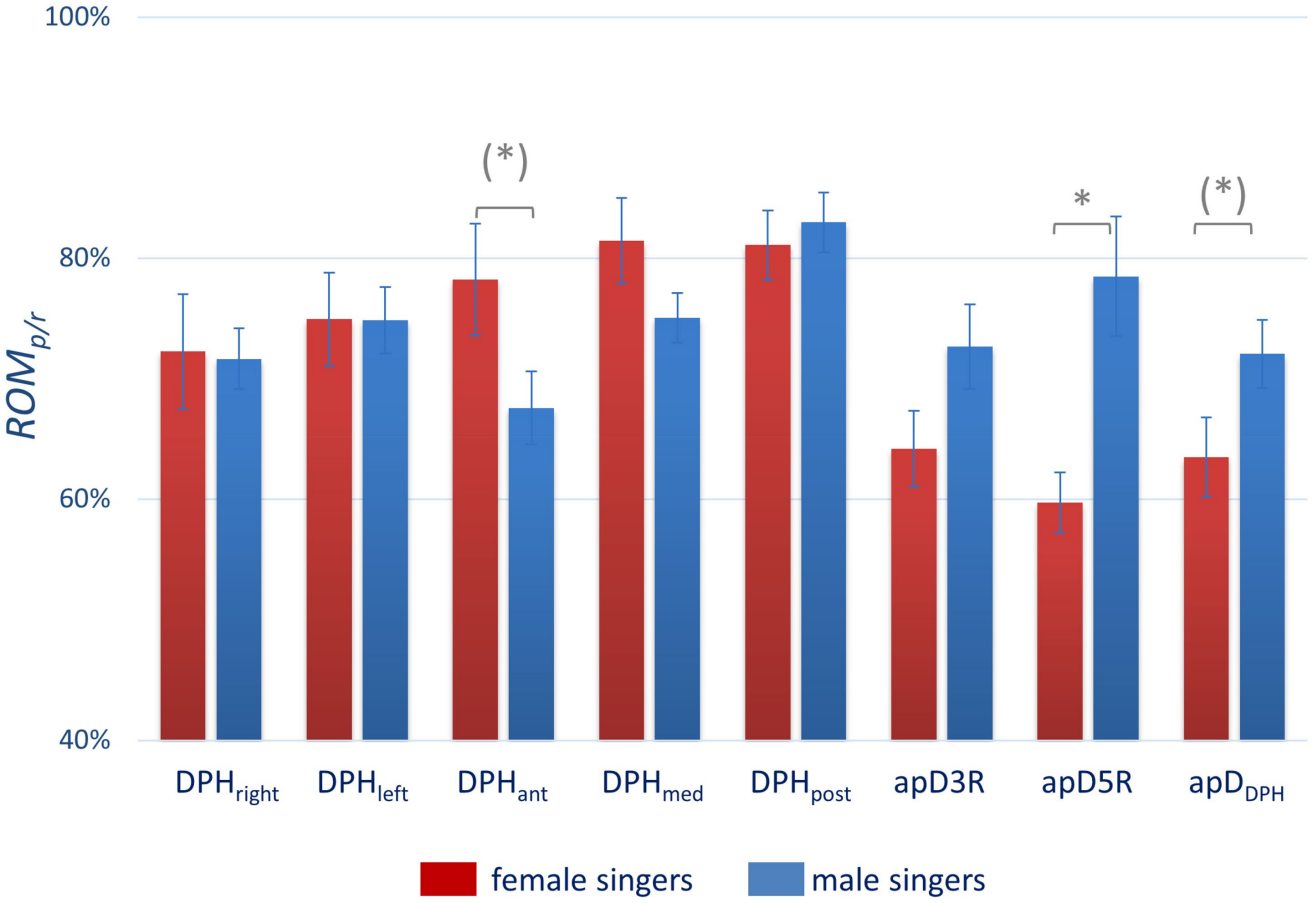
Significantly more thoracic movement in male and a tendency to more diaphragmatic movement in female subjects during maximal sustained phonation in relation to vital capacity respiration. The figure compares the phonatory movement range in relation to the respiratory movement range (ROM_p/r_) for male (blue) and female singers (red) for different locations. Significant differences are marked with *(*p* = <0.05) and tendencies with (*p = <0.10). All p-values are displayed in Table 5.

To further analyze the visual differences in Figures 4 and 5 and Supplementary Video S3, an individual quotient was calculated that represents the movement range (ROM) during maximum sustained phonation in relation to the individual VC respiration (ROM_r/p_). Larger ROM values indicate a larger utilization of the possible range of motion for the individual localization (see Table 5 and Figure 6). Respiratory movement of the thorax during phonation was stronger in male singers compared to females. In contrast, in female singers no statistically significant difference was found for ROM_r/p_.The ROM_r/p_ for the diaphragmatic movement in the anterior section (DPH_ant_) failed to reach statistical significance only marginally.

**Table 5:** Differences between male and female subjects’ phonatory movement range in relation to the respiratory movement range (ROM_p/r_), separately analyzed for each location.

Location	Statistical difference of DeltaROM_p/r_ in female vs. male singers
DPH_right_	*F*(1,49) = 0.01, *p* = 0.92, *ƞ*^2^ < 0.001
DPH_left_	*F*(1,49) < 0.001, *p* = 0.99, *ƞ*^2^ < 0.001
DPH_ant_	*F*(1,49) = 3.08, *p* = 0.09(*), *ƞ*^2^ = 0.06
DPH_med_	*F*(1,49) = 2.05, *p* = 0.16, *ƞ*^2^ = 0.01
DPH_post_	*F*(1,49) = 0.20, *p* = 0.65, *ƞ*^2^ = 0.004
apD_3R_	*F*(1,49) = 2.67, *p* = 0.11, *ƞ*^2^ = 0.05
apD_5R_	*F*(1,49) = 9.5, *p* = 0.003*, *ƞ*^2^ = 0.17
apD_DPH_	*F*(1,49) = 3.22, *p* = 0.08(*), *ƞ*^2^ = 0.06

Significant differences are marked with *(*p* = < 0.05) and tendencies with (*p = < 0.10).

## Discussion

In this study, sex-related differences of respiratory kinematics were analyzed during phonation using sagittal and coronal cross-sectional MRI data of the thorax of professional singers. While respiratory kinematics during phonation (see “Respiratory kinematics during sustained phonation“) were essentially consistent between male and female singers, male singers increased thoracic excursion during singing, which was not found in female singers. Additionally, distinct differences in movement velocities of individual anatomical regions became apparent that support the theory that there are certain differences between the phonatory strategy of female and male singers.

**Figure 7:**
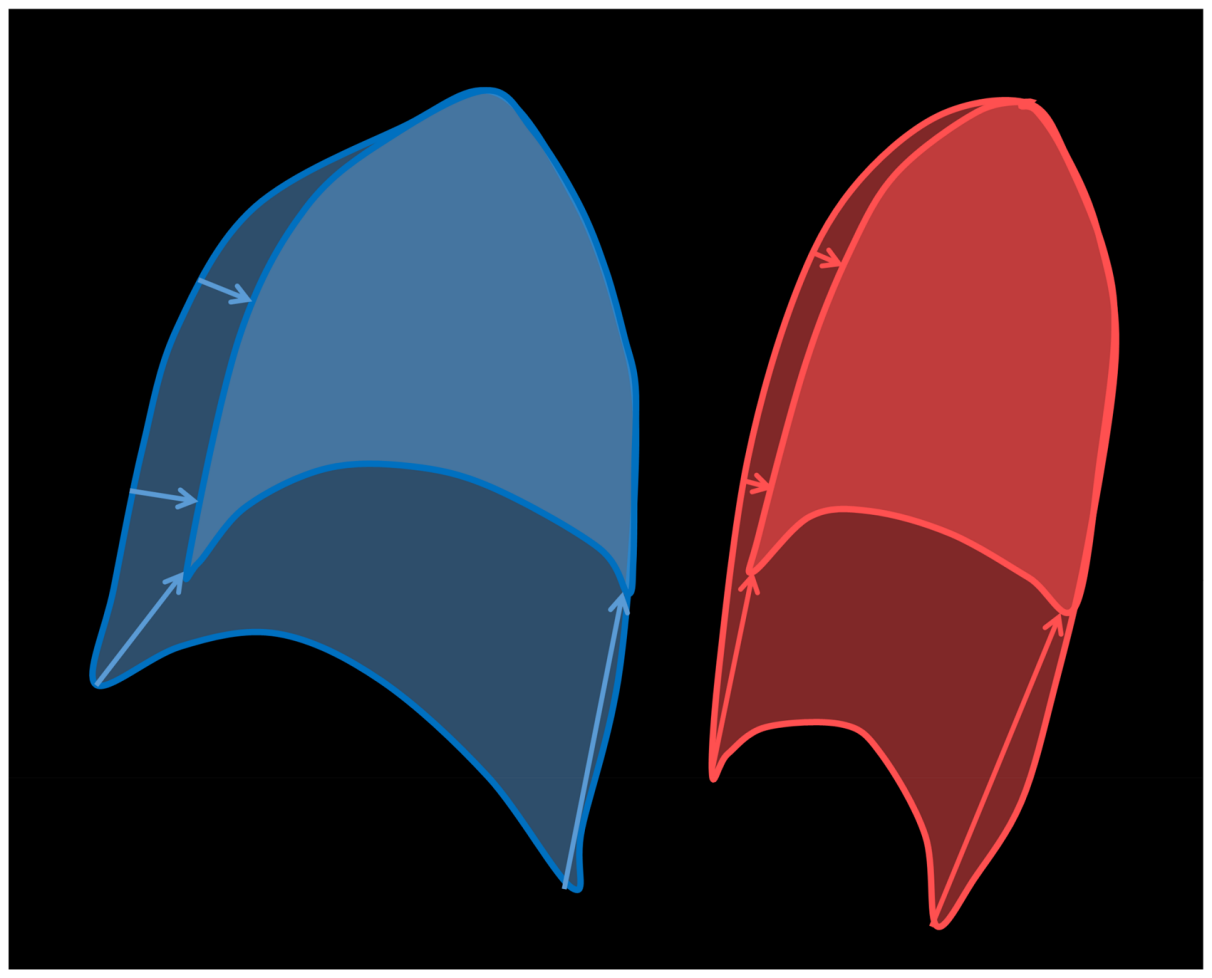
Illustration of a sagittal lung cross-section at the beginning (large area) and at the end (small area) of a phonation with drawing of the sex-specific movement ranges (arrows) for a male (blue) and a female (red) singer.

Breath support during singing is generally characterized by a simultaneous inspiratory activation of the DPH and thorax at the beginning of phonation to reduce *p_sub_* when recoil forces exceed the intended phonation threshold pressure (Sundberg, 1987). The DPH contraction during inspiration is then continuously relaxed during singing, at first in the posterior part of the DPH, while thorax and anterior DPH are held in an inspiratory position and thus move slower (Traser et al., 2017b). These kinematic movements are inverted at lower lung volumes when thorax movement accelerates driven by the increasing need for pressure generation to maintain the aspired *p_sub_* when negative recoil forces develop. These kinematics lead to the elliptic shape of the curves (Figure 2) observed in all subjects regardless of sex and comparable to observations of a previous pilot study (Traser et al., 2017b). Differences between female and male singers were found by inspection of separate anatomical locations (see section “Respiratory kinematics during sustained phonation”): in the upper thorax, inspiratory activation at the beginning of phonation was stronger in men, resulting in a lower motion velocity. In female singers the same happened for the anterior positioning of the DPH cupola (see Figures 3, 5). This is congruent with the observed predominance of thorax movement in relation to VC breathing: female singers elevated their thorax 20% less for maximum sustained phonation compared to male singers. Male singers initially show increased thoracic inspiratory activity to reduce the subglottic pressure, whereas female singers achieve this more through DPH activity [the anterior–posterior movement of the diaphragm cupola is a good measure of DPH contraction (Traser et al., 2020a)]. In addition to the predominance of thorax movement during the inspiration, male singers’ range of thorax motion was about 20% larger during phonation compared to that of female singers. Females, in contrast, tended to use a larger diaphragmatic movement range (especially in the anterior section) but results were not statistically significant. Watson et al. described that male singers initiated the majority of their vocal passages within the 80–100% VC range of rib-cage volume (Watson and Hixon, 1985) while female singers started at only 57–82% VC of their rib-cage volume (Watson et al., 1990). Their data is based on magnetometry, a technique that monitors anterior–posterior diameters of the thorax and abdomen but cannot image DPH motion directly. Instead, the anterior–posterior diameter of the abdomen was measured: Male singers initiated their vocal passages at abdominal volumes between 15 and 60% of VC compared to 53–86% in female singers. Even if this value is not directly transferable to DPH movement (as it is also affected by abdominal wall contraction), it could be an indication that a preference for DPH movement in females and thorax movements in males also occurred in Watsons’ data. Still, the authors stated no significant difference between the respiration of male and female singers and only a weak correlation between the male subjects’ self-perception of their breathing to the breathing measurements (Watson and Hixon, 1985). However, their data and this work are in good agreement with a questionnaire-based survey of Scott McCoy, who found that females concentrated their breathing efforts in lower parts of the body than men (hypo-vs. epi-gastric regions; McCoy, 2005). A stronger breathing effort in the epigastric region could be caused by pronounced thoracic movements, while a focus in the hypogastric region could be more related to the DPH-induced movement to the abdominal organs and corresponding abdominal wall regulation. In the voice pedagogic literature McCoy finds two pedagogic concepts about breathing for singing: the first uses a method that is mainly described by men, which sees breathing as an activity centered on and controlled by actions of the thorax, epigastrium and/or middle-back. In contrast, the second concept (mainly described by women) sees breathing as an activity of the lower thorax, hypogastric region, lower back and pelvis (McCoy, 2005). These results are in line with our previous work on pulmonary ventilation during singing (Traser et al., 2017a). Here, the location of the maximum change in ventilation showed a greater anterior – posterior variation in men, while it remained largely constant in females which might be originated from a larger range of rib-cage movement in men. In summary, these descriptions seem to fit the theory of sex-related differences in respiratory control for singing. Figure 7 illustrates a typical sagittal lung cross section of a female and male singer at the beginning and end of phonation. The arrows emphasize the discussed sex-specific range of motion.

## Discussion of potential origins of the described preferences in respiratory behavior between male and female singers

### Sex related anatomical/physiological differences of respiratory system

Observed differences could be related to differences in the respiratory anatomy/physiology between males and females: On average, men are taller, have bigger lungs and larger RC dimensions than women and thus higher vital capacity (Thurlbeck, 1982; Bellemare et al., 2003; Torres-Tamayo et al., 2018). However, during respiration a greater contribution of inspiratory rib-cage motion to tidal volume displacement can be found in females than males. It has been discussed that the greater incline angle of the ribs in females may put the inspiratory rib-cage muscles at a better mechanical advantage (Bellemare et al., 2003; Romei et al., 2010; it is speculated that the greater contribution of the rib-cage in women may be propaedeutic for the functional adaptation to the hormonal and anatomical changes induced by pregnancy). Still, sex-related differences of operational lung volumes during exercise were attributable to differences related to lung size as well as differences in the compartmental analysis of the kinematics of the entire chest wall (Vogiatzis et al., 2005).

Thus, even if women might have a constitutionally more effective RC system, the presented data and reviewed literature could be taken as indications that female singers developed a pronounced DPH movement during singing phonation while men emphasized their RC movement. It could therefore be speculated that both sexes expand their capacities during singing training in the section that is less advantaged by nature. Nevertheless, since men and women in the described cohort differed significantly with respect to body size, it was not possible to further determine whether the cause of the described differences was their body configuration or control of phonation. Additionally, interactions between breathing kinematics and voice source also have been taken into account when the potential benefits of predominant control of voice production from the DPH or thorax is assessed.

### Sex related anatomical/physiological differences of laryngeal functions

The male vocal folds are longer and thicker with larger vibrational amplitudes (Titze, 1989). This leads to a stronger and quicker glottal closure in men combined with higher airflow rates compared to females (Sulter and Wit, 1996). Classically trained female singers phonate proportionally more in a register function which is characterized by a reduction of the vibrating mass (also called head or falsetto register or M2). It stands in contrast to full vibration of the vocal folds also called (chest or modal register, M1), which is predominantly used in males (Henrich et al., 2005; Sundberg, 2018). OQ during singing is reported to be higher in female compared to male singers (Sulter and Wit, 1996), which is corroborated in the presented data. The OQ was found to remain essentially constant with *f*_o_ in male singers while it increased with pitch in females (Howard, 1995), which is congruent to our data. As M2, in contrast to M1, was described to correlate well with OQ, the use of these different mechanisms seems to be responsible for the difference between male and female singers in our data as well.

*Via* the so-called tracheal pull, glottal closure is related to the lung volume (Thomasson, 2003). It was previously shown that high DPH activity corresponds to a lower laryngeal position that increases laryngeal abductory forces [as the cricothyroid muscle is more activated with increasing tracheal pull which generates glottal abduction (Leanderson et al., 1984; Sundberg et al., 1989; Iwarsson and Sundberg, 1998)]. This relation was less expressed in a study on professional female singers by Ternström et al., probably as they learn to control these parameters independently (Ternström et al., 2018). In male professional singers, DPH activation was found to relate to so-called “flow phonation,” which is characterized by an increase in glottal airflow and thus OQ compared to normal phonation (Leanderson et al., 1987).

Consideration of the presented data in the context of type of phonation, leads to the question of whether a particular kind of breath support (more thorax or more DPH based) might be more or less beneficial for the intended vocal fold vibration. Thus, it might not be a coincidence that DPH predominance was typical for female singers of our cohort, as their vocal fold vibration (in the Western style classical context) is characterized by lower airflow rates and smaller amplitude of vibration. Interestingly, a reduction of respiratory thorax movement has also been observed when singers used the so-called DPH-co-contraction technique (Sundberg et al., 1989). Here a conscious overpressure from the abdominal wall muscles is actively reduced by DPH contraction during phonation. In contrast, a more thorax related respiratory pattern was utilized by male singers whose vocal fold vibratory patterns are generally characterized by higher glottal airflow, larger amplitude and thus maximum flow declination rate (MFDR; Sulter and Wit, 1996). In this context it is very interesting to see that in a single subject study, a professional female singer increased her thorax inspiration by about 20% when she switched from Western classically style to belting (Traser et al., 2020b), a contemporary music singing style which is characterized by a higher degree of glottal adduction (Sundberg et al., 1993) and higher vertical laryngeal position (Echternach et al., 2014). In the next step also the interaction between the vertical laryngeal position and the acoustics of the vocal tract shall be discussed.

### Sex related anatomical/physiological differences of the vocal tract

The vocal tract is longer in males compared to females (Sundberg, 1987). Elevation of the larynx has an acoustical effect on the voice by shortening the resonance cavity, and this affects all formant frequencies (Sundberg, 1987). A low larynx position is desired in some fields of Western classical singing, e.g., when male singers want to boost energy in the frequency region of the singers’ formant (Sundberg, 1987). In contrast larynx elevation might be actively induced when female singers are adjusting the first two formants to their fundamental frequency to increase loudness [formant tuning; (Weiss et al., 2001)] as well as for some singing styles in contemporary music, e.g., belting (Echternach et al., 2014). Generally, the larynx has been found to elevate with pitch in untrained subjects (Shipp, 1975; Traser et al., 2014), but less so in trained singers (Shipp and Izdebski, 1975; Traser et al., 2013). It could therefore be speculated that, for men (who’s timbre is more easily influenced due to lower *f*_o_ and thus closer partials), it would be more relevant to keep the larynx position steady, although some elevation is noted to occur in upwards scale singing even in highly trained tenors (Echternach et al., 2010). This technique could be supported through predominant thorax respiration, which would reduce tracheal pull compared to DPH activation. In contrast, in highly trained female singers even significant elevation of the larynx with pitch (Shipp et al., 1987; Weiss et al., 2001) and increased loudness (Mainka et al., 2021) has been observed. A more pronounced DPH activation could lead to a higher variability in larynx position, which is acceptable when singing in a high pitch range, where the formant frequencies are difficult to detect (because thousands of hertz lie between partials; Shipp et al., 1987).

## Limitations

In this study only 12 classically trained singers were included – even though this is currently the largest cohort with an MRI-based analysis, the group is too small to exclude a group size effect. All data were first included in a rmANOVA test using covariates to test for the effect of confounding variables. In a second step individual calculations were performed for separated locations. Still, the problems of non-independence and heteroscedasticity of data remain and could confound the statistical analysis. Therefore, the statistical power of the data is limited. Still, it was not possible to include more professional singers to enrich the statistical power. In our opinion, including statistics in this manuscript is still an advantage in order to enable an initial classification of the results. However, the presented results need to be confirmed in a larger study.

In this study, beside one baritone, only sopranos and tenors were included. The recruitment of the singers was done through personal contacts, so the composition of the singers’ voice classification was random. Thus it cannot be excluded, that the difference is only a typical behavior of sopranos vs. tenors and not female vs. male singers. Again, an expansion of the data with more subjects is necessary to further illuminate the question of whether there are also differences related to voice classification.

In this study measurements were taken in a supine body position due to the use of a clinical horizontal-bore MRI system. Lying on the back, gravity exerts a force on the lungs and thus influences *p_sub_* so that the demand for raising *p_sub_* by muscular means is smaller. Studies on posture-related differences during respiration showed that the DPH-motion in the supine position was significantly greater than that in the upright position (Takazakura et al., 2004) and the anteroposterior diameters of the chest wall motion were significantly lower in the supine position (Takashima et al., 2017). For untrained subjects Hixon et al. described a substantial change in respiratory kinematics during sustained utterances between upright and supine position (in supine position the inspiratory braking was solely performed by DPH without support by the inspiratory thorax muscles, which are normally primarily involved in the upright position (Hixon et al., 1976). Such a fundamental change in respiratory kinematics could not be confirmed in professional singers (Traser et al., 2021): The diaphragm was cranially displaced in supine position for both singing and breathing and its motion range increased. Still, regardless of body position, singers maintained their general principles of respiratory kinematics with combined diaphragm and thorax muscle activation for breath support. This was achieved by expanding their chest an additional 20% during inspiration when singing in the supine position but not for sole breathing (this stands in contrast to the reduction in thoracic motion for untrained subjects in supine position in the study of Takashima et al., 2017).

However, as both male and female singers were in supine position, it is unlikely that differences between the respiratory kinematics of men and women are posture-related. As all subjects were trained singers, they are used to singing in different body positions and have very consistent breathing strategies (Thomasson and Sundberg, 1999).

Another influencing factor might be the body configuration and weight of the singers. A previous study analyzed the correlation of body type and breathing tendencies by video analysis in young female singing students (Cowgill, 2009). The author found that overweight singers tended to breathe lower (movement in the umbilicus area), while muscular singers predominantly moved the rib-cage area and lean (and long) singers the lateral chest area. Another plethysmography study in untrained subjects did not find any between-group differences in respiratory motions for body type (Manifold and Murdoch, 1993). As obese people have a higher abdominal inertance, their body weight might affect breathing in a non-linear way. Nevertheless, as male and female singers did not differ concerning their BMI in the analyzed cohort, a large effect of body weight seems unlikely in the presented data. The male singers in this cohort were significantly taller than the females. Thus, to avoid scaling effects, relative distances were calculated and compared. Unfortunately, due to the small sample size no further subgroup analysis could be performed to differentiate between sex-or body height related differences. Still different body geometries, size/weight of abdomen or breasts could have varying degrees of impact on the singers especially in supine position, which cannot be excluded due to the limited sample size.

## Conclusion

The presented data showed no sex-related difference concerning basic respiratory kinematics during phonation. However, male singers increased their thoracic contribution while singing, a difference which was not found in female singers, who tended to increase their DPH breathing during phonation. As DPH activation is associated with an increase in tracheal pull and thus activation of cricothyroid muscle with lower laryngeal position and glottal abduction, there might be a relation of respiratory kinematics with the intended vocal fold vibration as well as vocal tract configuration and thus resonance strategies (Leanderson et al., 1984; Sundberg et al., 1989; Iwarsson and Sundberg, 1998). However, other contributing factors like body configuration, the subject’s Fach (e.g., lyric vs. dramatic voices, different repertoire) and genre (classical vs. contemporary music style) might also be relevant and due to limited sample size no final conclusion can be drawn from the presented data. Nevertheless, data indicate that the sex of the singer influences the predominance for different respiratory strategies during singing. When considering vocal pedagogical constellations with mixed-sex teacher-student combinations, it might be advisable to consider these aforementioned differences in the singing training.

## Author’s note

Effective breath control is considered to be an important factor in singing voice production, but descriptions of how this is accomplished vary widely. In addition to pedagogical concepts, gender differences could also play a role here. In order to enhance understanding of differences in respiratory kinematics between male and female singers, sagittal and coronal cross-sectional images of the lung were analyzed using dynamic MRI in 12 professional singers. While basic movement patterns of breath support were essentially consistent between male and female singer, male singers showed increased thoracic breathing compared to female singers. In contrast, female singers tended to increase their diaphragm movement during phonation. The presented data shows the need for an individual approach for teaching of breath support strategies during singing voice training.

## Data availability statement

The raw data supporting the conclusions of this article will be made available by the authors, without undue reservation.

## Ethics statement

The studies involving human participants were reviewed and approved by Medical Ethics Committee of the University of Freiburg, Germany. The patients/participants provided their written informed consent to participate in this study.

## Author contributions

LT initiated the study, made measurements, analyzed data and wrote the paper. CS made measurements, analyzed data. ACÖ made measurements. FB made measurements. MB and BR conducted and supervised research. ME analyzed data, wrote the paper and supervised research. All authors contributed to the article and approved the submitted version.

## Funding

The article processing charge was funded by the Baden-Wuerttemberg Ministry of Science, Research and Art and the University of Freiburg in the funding program Open Access Publishing.

## Conflict of interest

The authors declare that the research was conducted in the absence of any commercial or financial relationships that could be construed as a potential conflict of interest.

## Publisher’s note

All claims expressed in this article are solely those of the authors and do not necessarily represent those of their affiliated organizations, or those of the publisher, the editors and the reviewers. Any product that may be evaluated in this article, or claim that may be made by its manufacturer, is not guaranteed or endorsed by the publisher.

## Footnotes

^1^The Bunch and Chapman taxometrie offers criteria for determining the classification of singers in research. It suggests a system in which scientific research related to singing is based on a quantifiable singing standard. It includes nine singer categories based on proven performance achievement. These categories range from superstar (household name) to local community and amateur singers with com-prehensive subcategories under each topic for each type of singer, that is, opera, pop, jazz, etc.

## References

[ref1] Baken R. J. Orlikoff R. F. (2000). Clinical measurement of speech and voice. San Diego: Singular Publishing Group, Thomson Learning.

[ref2] Bellemare F. Jeanneret A. Couture J. (2003). Sex differences in thoracic dimensions and configuration. Am. J. Respir. Crit. Care Med. 168, 305–312. doi: 10.1164/rccm.200208-876OC, PMID: 12773331

[ref3] Bunch M. Chapman J. (2000). Taxonomy of singers used as subjects in scientific research. J. Voice 14, 363–369. doi: 10.1016/S0892-1997(00)80081-8, PMID: 11021503

[ref4] Cowgill J. G. (2009). Breathing for singers: a comparative analysis of body types and breathing tendencies. J. Sing. 66:141.

[ref5] Echternach M. Popeil L. Traser L. Wienhausen S. Richter B. (2014). Vocal tract shapes in different singing functions used in musical Theater singing - a pilot study. J. Voice 28, 653.e1–653.e7. doi: 10.1016/j.jvoice.2014.01.011, PMID: 24810998

[ref6] Echternach M. Sundberg J. Markl M. Richter B. (2010). Professional opera tenors’ vocal tract configurations in registers. Folia Phoniatr. Logop. 62, 278–287. doi: 10.1159/000312668, PMID: 20588050

[ref7] Griffin B. Woo P. Colton R. Casper J. Brewer D. (1995). Physiological characteristics of the supported singing voice. A preliminary study. J. Voice 9, 45–56. doi: 10.1016/S0892-1997(05)80222-X, PMID: 7757150

[ref8] Henrich N. D’Alessandro C. Doval B. Castellengo M. (2005). Glottal open quotient in singing: measurements and correlation with laryngeal mechanisms, vocal intensity, and fundamental frequency. J. Acoust. Soc. Am. 117, 1417–1430. doi: 10.1121/1.1850031, PMID: 15807029

[ref9] Hixon T. J. Hoit J. (2005). Evaluation and Management of Speech Breathing Disorders Priciples and Methods. Tuscon, Arizona: Redington.

[ref10] Hixon T. J. Mead J. Goldman M. D. (1976). Dynamics of the chest wall during speech production: function of the thorax, rib cage, diaphragm, and abdomen. J. Speech Hear. Res. 19, 297–356. doi: 10.1044/jshr.1902.297, PMID: 135885

[ref11] Howard D. M. (1995). Variation of electrolaryngographically derived closed quotient for trained and untrained adult female singers. J. Voice 9, 163–172. doi: 10.1016/S0892-1997(05)80250-4, PMID: 7620539

[ref12] Howard D. M. Lindsey G. A. Allen B. (1990). Toward the quantification of vocal efficiency. J. Voice 4, 205–212. doi: 10.1016/S0892-1997(05)80015-3

[ref13] Iwarsson J. Sundberg J. (1998). Effects of lung volume on vertical larynx position during phonation. J. Voice 12, 159–165. doi: 10.1016/S0892-1997(98)80035-0, PMID: 9649071

[ref14] Leanderson R. Sundberg J. von Euler C. (1984). Effect of diaphragm activity on phonation during singing. STL-QPSR 25, 001–010.

[ref15] Leanderson R. Sundberg J. von Euler C. (1987). Role of diaphragmatic activity during singing: a study of transdiaphragmatic pressures. J. Appl. Physiol. 62, 259–270. doi: 10.1152/jappl.1987.62.1.259, PMID: 3558185

[ref16] Mainka A. Platzek I. Klimova A. Mattheus W. Fleischer M. Mürbe D. (2021). Relationship between epilarynx tube shape and the radiated sound pressure level during phonation is gender specific. Logop. Phoniatr. Vocology 13, 1–13. doi: 10.1080/14015439.2021.1988143, PMID: 34644212

[ref17] Manifold J. A. Y. Murdoch B. E. (1993). Speech breathing in young adults: effect of body type. J. Speech Hear. Res. 36, 657–671. doi: 10.1044/jshr.3604.657, PMID: 8377479

[ref18] McCoy S. (2005). Breath management: gender-based differences in classical singers. Folia Phoniatr. Logop. 57, 246–254. doi: 10.1159/000087078, PMID: 16280628

[ref19] Özen A. C. Traser L. Echternach M. Dadakova T. Burdumy M. Richter B.. (2015). Ensuring safety and functionality of electroglottography measurements during dynamic pulmonary MRI. Magn. Reson. Med. 76, 1629–1635. doi: 10.1002/mrm.26037 26599237

[ref20] Romei M. Mauro A. L. D’Angelo M. G. Turconi A. C. Bresolin N. Pedotti A.. (2010). Effects of gender and posture on thoraco-abdominal kinematics during quiet breathing in healthy adults. Respir. Physiol. Neurobiol. 172, 184–191. doi: 10.1016/j.resp.2010.05.018, PMID: 20510388

[ref21] Shipp T. (1975). Vertical laryngeal position during continuous and discrete vocal frequency change. J. Speech. Lang. Hear. Res. 18, 707–718. doi: 10.1044/jshr.1804.707 1207101

[ref22] Shipp T. Guinn L. Sundberg J. Titze I. (1987). Vertical laryngeal position—research findings and their relationship to singing. J. Voice 1, 220–222. doi: 10.1016/S0892-1997(87)80003-6, PMID: 26776950

[ref23] Shipp T. Izdebski K. (1975). Vocal frequency and vertical larynx positioning by singers and nonsingers. J. Acoust. Soc. Am. 58, 1104–1106. doi: 10.1121/1.380776, PMID: 1194561

[ref24] Sonninen A. Laukkanen A.-M. Karma K. Hurme P. (2005). Evaluation of support in singing. J. Voice 19, 223–237. doi: 10.1016/j.jvoice.2004.08.003, PMID: 15907437

[ref25] Sorichter S. (2009). Lung function. Radiologe 49, 676–686. doi: 10.1007/s00117-009-1877-0, PMID: 19693617

[ref26] Sulter A. M. Wit H. P. (1996). Glottal volume velocity waveform characteristics in subjects with and without vocal training, related to gender, sound intensity, fundamental frequency, and age. J. Acoust. Soc. Am. 100, 3360–3373. doi: 10.1121/1.416977, PMID: 8914317

[ref27] Sundberg J. (1987). The science of the singing voice. Dekalb, Illinois: Northern Illinois University Press.

[ref28] Sundberg J. (2018). Flow glottogram and subglottal pressure relationship in singers and untrained voices. J. Voice 32, 23–31. doi: 10.1016/j.jvoice.2017.03.024, PMID: 28495328

[ref29] Sundberg J. Gramming P. Lovetri J. (1993). Comparisons of pharynx, source, formant, and pressure characteristics in operatic and musical theatre singing. J. Voice 7, 301–310. doi: 10.1016/S0892-1997(05)80118-3, PMID: 8293062

[ref30] Sundberg J. Leanderson R. von Euler C. (1989). Activity relationship between diaphragm and cricothyroid muscles. J. Voice 3, 225–232. doi: 10.1016/S0892-1997(89)80004-9, PMID: 16645190

[ref31] Takashima S. Nozoe M. Mase K. Kouyama Y. Matsushita K. Ando H. (2017). Effects of posture on chest-wall configuration and motion during tidal breathing in normal men. J. Phys. Ther. Sci. 29, 29–34. doi: 10.1589/jpts.29.29, PMID: 28210033 PMC5300799

[ref32] Takazakura R. Takahashi M. Nitta N. Murata K. (2004). Diaphragmatic motion in the sitting and supine positions: healthy subject study using a vertically open magnetic resonance system. J. Magn. Reson. Imaging 19, 605–609. doi: 10.1002/jmri.20051, PMID: 15112310

[ref33] Ternström S. D’Amario S. Selamtzis A. (2018). Effects of the lung volume on the electroglottographic waveform in trained female singers. J. Voice. 34:485.e1-485.e21. doi: 10.1016/j.jvoice.2018.09.006 30337119

[ref34] Thomasson M. (2003). Effects of lung volume on the glottal voice source and the vertical laryngeal position in male professional opera singers. TMH-QPSR 45, 001–009.

[ref35] Thomasson M. Sundberg J. (1999). Consistency of phonatory breathing patterns in professional operatic singers. J. Voice 13, 529–541. doi: 10.1016/S0892-1997(99)80008-3, PMID: 10622519

[ref36] Thurlbeck W. M. (1982). Postnatal human lung growth. Thorax 37, 564–571. doi: 10.1136/thx.37.8.564, PMID: 7179184 PMC459376

[ref37] Titze I. R. (1989). Physiologic and acoustic differences between male and female voices. J. Acoust. Soc. Am. 85, 1699–1707. doi: 10.1121/1.397959, PMID: 2708686

[ref38] Torres-Tamayo N. García-Martínez D. Lois Zlolniski S. Torres-Sánchez I. García-Río F. Bastir M. (2018). 3D analysis of sexual dimorphism in size, shape and breathing kinematics of human lungs. J. Anat. 232, 227–237. doi: 10.1111/joa.12743, PMID: 29148039 PMC5770305

[ref39] Traser L. Burdumy M. Richter B. Vicari M. Echternach M. (2013). The effect of supine and upright position on vocal tract configurations during singing —a comparative study in professional tenors. J. Voice 27, 141–148. doi: 10.1016/j.jvoice.2012.11.002, PMID: 23380394

[ref40] Traser L. Burdumy M. Richter B. Vicari M. Echternach M. (2014). Weight-bearing MR imaging as an option in the study of gravitational effects on the vocal tract of untrained subjects in singing phonation. PLoS One 9:e112405. doi: 10.1371/journal.pone.0112405, PMID: 25379885 PMC4224454

[ref41] Traser L. Burk F. Özen A. C. Burdumy M. Bock M. Blaser D.. (2020a). Respiratory kinematics and the regulation of subglottic pressure for phonation of pitch jumps – a dynamic MRI study. PLoS One 15:e0244539. doi: 10.1371/journal.pone.0244539, PMID: 33382744 PMC7775092

[ref42] Traser L. Knab J. Echternach M. Fuhrer H. Richter B. Buerkle H.. (2017a). Regional ventilation during phonation in professional male and female singers. Respir. Physiol. Neurobiol. 239, 26–33. doi: 10.1016/j.resp.2017.01.006, PMID: 28109943

[ref43] Traser L. Özen A. C. Burk F. Burdumy M. Bock M. Richter B.. (2017b). Respiratory dynamics in phonation and breathing—a real-time MRI study. Respir. Physiol. Neurobiol. 236, 69–77. doi: 10.1016/j.resp.2016.11.007, PMID: 27871890

[ref44] Traser L. Rummel S. Schwab C. Özen A. C. Bock M. Echternach M.. (2020b). “Diaphragm and rip cage movements during phonation of professional singers of different genres – a dynamic MRI study,” in Proceedings of the ICVBP (Genoble).

[ref45] Traser L. Schwab C. Burk F. Özen A. C. Burdumy M. Bock M.. (2021). The influence of gravity on respiratory kinematics during phonation measured by dynamic magnetic resonance imaging. Sci. Rep. 11, 1–13. doi: 10.1038/s41598-021-02152-y 34824315 PMC8617256

[ref46] Vogiatzis I. Aliverti A. Golemati S. Georgiadou O. Lomauro A. Kosmas E.. (2005). Respiratory kinematics by optoelectronic plethysmography during exercise in men and women. Eur. J. Appl. Physiol. 93, 581–587. doi: 10.1007/s00421-004-1249-4, PMID: 15578206

[ref47] Watson P. J. Hixon T. J. (1985). Respiratory kinematics in classical (opera) singers. J. Speech Hear. Res. 28, 104–122. doi: 10.1044/jshr.2801.104, PMID: 3157022

[ref48] Watson P. J. Hixon T. J. Stathopoulos E. T. Sullivan D. R. (1990). Respiratory kinematics in female classical singers. J. Voice 4, 120–128. doi: 10.1016/S0892-1997(05)80136-5, PMID: 27159498

[ref49] Weiss R. Brown W. S. Moris J. (2001). Singer’s formant in sopranos: fact or fiction? J. Voice 15, 457–468. doi: 10.1016/S0892-1997(01)00046-7, PMID: 11792022

