# Supplementary material for: Differences of respiratory kinematics in female and male singers – A comparative study using dynamic magnetic resonance imaging
Source: Front Psychol. 2022 Dec 5;13:844032. doi: 10.3389/fpsyg.2022.844032 (PMC9760878; doi:10.3389/fpsyg.2022.844032)
Supplement: Supplementary file 1 [file Table_1.DOCX]

| **Task** | **OQ male vs. female** | ***p_sub_* male va. female** | **SPL male vs. female** |
| --- | --- | --- | --- |
| **P1mf** | F (1,12) = .02, p= .89 ƞ^2^= .14 | F (1,11) = .59, p= .43 ƞ^2^= .06 | F (1,11) = 1.58, p= .24 ƞ^2^= .002 |
| **P2mf** | F (1,12) = 12.52, p= .006 ƞ^2^= .58 | F (1,11) = .79, p= .40 ƞ^2^= .08 | F (1,11) = .01, p= .92 ƞ^2^= .001 |
| **P3mf** | F (1,12) = 8.34, p= .02 ƞ^2^= .46 | F (1,11) = 1.84, p= .21 ƞ^2^= .17 | F (1,11) = 4.22, p= .07 ƞ^2^= .32 |
| **P2pp** | F (1,12) = 2.40, p= .16 ƞ^2^= .21 | F (1,11) = .84, p= .38 ƞ^2^= .09 | F (1,11) = .995, p= .35 ƞ^2^= .10 |
| **P2ff** | F (1,12) = 18,76, p= .002 ƞ^2^= .82 | F (1,11) = 1.09, p= .32 ƞ^2^= .11 | F (1,11) = .07, p= .79 ƞ^2^= .01 |

**Supplement Table S1: Difference between male and female singers for open quotient (OQ), subglottic pressure (p_sub_) and sound pressure level SPL calculated for each task separately. Statistically significant differences are marked with grey.**

| **Location** | **Difference in curve progression in M_1-5_ male vs. female** |
| --- | --- |
| **DPH_right_** | F (4,59) = .65, p= .55 ƞ^2^= .003 |
| **DPH_left_** | F (4,59) = 1.33, p= .27 ƞ^2^= .005 |
| **DPH_ant_** | F (4,59) = 2.20, p= .40 ƞ^2^= .02 |
| **DPH_med_** | F (4,59) = 4.26, p= .18 ƞ^2^= .03 |
| **DPH_post_** | F (4,59) = 1.83, p= .16 ƞ^2^= .03 |
| **apD_3R_** | F (4,59) = 3.10 p= .04 ƞ^2^= .05 |
| **apD_5R_** | F (4,59) =2.08 p= .11 ƞ^2^= .03 |
| **apD_DPH_** | F (4,59) = 3.57 p= .02 ƞ^2^= .05 |

**Supplement Table S2: Difference in curve progression of normalized sustained phonation between male and female singers calculated for each location separately. Statistically significant differences are marked with grey.**

**Supplement Video S3: Exemplary film of one female and one male subjects’ respiratory movements during phonation of P2mf (440Hz for female and 200Hz for male subject). The movement time was normalized to make it comparable.**
